# *Cryptosporidium parvum* and *Cryptosporidium andersoni* infection in naturally infected cattle of northwest Iran

**Published:** 2014

**Authors:** Yousef Mirzai, Mohammad Yakhchali, Karim Mardani

**Affiliations:** 1 *Department of Pathobiology, Faculty of Veterinary Medicine, UrmiaUniversity, Urmia, Iran; *; 2 *Department of Food Hygiene and Quality Control, Faculty of Veterinary Medicine, Urmia University, Urmia, Iran.*

**Keywords:** 18S rRNA gene, *Cryptosporidium andersoni*, *Cryptosporidium parvum*, Iran

## Abstract

The protozoan intestinal parasite *Cryptosporidium* commonly infects cattle throughout the world and Iran. The present study was undertaken to determine the abundance and associated risk factors of *Cryptosporidium* infection in cattle herds of northwestern Iran. A total number of 246 fecal samples from 138 (56.1%) diarrheic (D) and 108 (43.9%) non-diarrheic (ND) cattle were randomly collected and examined by fecal smears stained with Ziehl-Neelsen. For molecular specification, DNA was extracted from collected *Cryptosporidium* oocysts and a fragment of 1325 bp in size from 18S rRNA gene was amplified. The overall prevalence of *Cryptosporidium *infection was 22.3% (55/246). The prevalence of *Cryptosporidium* infection in examined calves less than 6 month-old was significantly higher than adult cattle. *C. parvum* and *C. andersoni* were identified in 20.3% (50/246) and 2.03% (5/246) of examined cattle, respectively. The highest prevalence of *C. parvum *infection was found in D calves < 6 month-old (13.4%, 33/246), while *C. andersoni* was only detected in ND cattle (8.9%, 22/246). There was significant difference in the prevalence between male than female cattle. There was no significant difference between prevalence and seasons of investigation. It was concluded that *C. parvum* was the prevalent species in younger animals compared to older ones as a potentially zoonotic agent in the region.

## Introduction

*Cryptosporidium* an obligate intracellular protozoan parasite is a frequent cause of intestinal, gastric or respiratory cryptosporidiosis in a wide range of animals and humans hosts worldwide.^1^ Some of the zoonotic *Cryptosporidium *species usually causes self-limiting diarrhea in humans and animals. Cryptosporidiosis was reported for the first in 1971,^2^ as well as from Iranian cattle in 1984,^3^ and increasingly in a range of hosts.^1^^,^^4^^-^^7^

Over the past two decades, cattle have been identified as a common reservoir host for *Cryptosporidium* species. Currently, 20 different species of *Cryptosporidium* has been reported which *C. parvum, C. bovis, C. ryanae*,* Cryptosporidium* deer-like genotype, and* C. andersoni* are considered as cattle adapted.^8^ Of those, the intestinal specie *C. parvum* has zoonotic potential and is a frequent cause of human cryptosporidiosis.^9^^,^^10 ^In terms of economic losses, *C. parvum* is considered as the most important species among cryptosporidial agents.^11^ *Cryptosporidium andersoni* as an abomasal parasite has been associated with reduced milk yield in dairy cattle and decreased weight gain in post weaned calves.^12^

According to Xiao *et al.*, a high diversity of *Cryptosporidium* genus based on several molecular markers, the 18S rRNA gene, has been shown by multilocus DNA analysis.^10^ In Iran, molecular detection of *Cryptosporidium* in humans has been undertaken by several researchers.^11^^,^^13^^,^^14^ While, to date, the molecular study for confirming cattle *Cryptosporidium* infection has not been investigated in this part of Iran. Additionally, the nationwide epidemiological survey is essential for the exact knowledge of infection status of *C. parvum *and *C. andersoni* in the country. Therefore, it is important to determine bovine *Cryptosporidium *infection and major risk factors in order to screen *C. parvum* and *C. andersoni* harboring in cattle herds of northwestern Iran.

## Materials and Methods

**Fig. 1:**
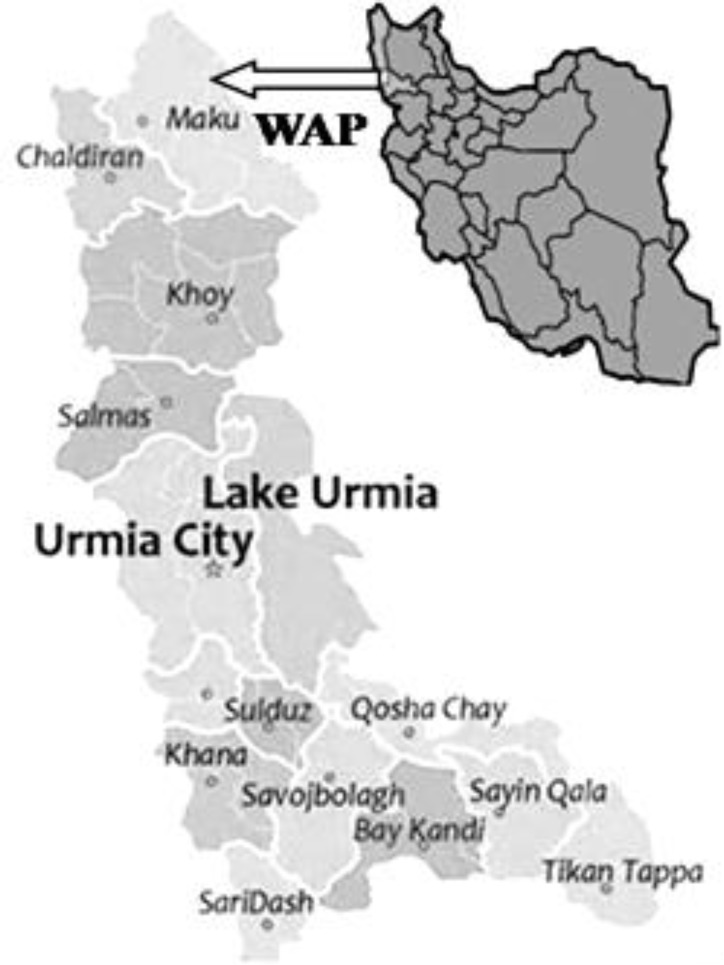
Map of West Azarbaijan province (WAP) showing the places with samples of cattle herd examined for infection

**Study area. **The study area (West Azarbaijan province, WAP) is located in northwest of Iran with two rainy seasons, the first from March to May and the second in October-November. The study was carried out in the mountainous, mountainside and plain areas of Urmia suburb covering 355 villages that fall within a radius of 20 to 125 km of the city center (Fig. 1). According to the Iranian Veterinary Organization, an average population of eight million cattle is distributed in Iran; the WAP has approximately 6.1% of these cattle.^15^

**Animals.** During the course of the study (September 2010 to August 2011), a total number of 101 cattle and 145 calves from 16 herds (375 cattle) were randomly selected from cattle herds in Urmia suburban of WAP. The herds examined were raised following traditional husbandry practices, with animals being mainly cross-breeds and indigenous crossbred. The major risk factors for *Cryptosporidium *infection were host, *Cryptosporidium *species, and environmental factors (Table 1). The cattle were divided into four age groups, numbered and subjected to clinical examination. The age was estimated on the basis eruption of permanent incisor teeth.^16^ The consistency (Fc) of fecal specimens (D or ND) were recorded. The sample size for determining prevalence was estimated based on the formula (expected prevalence 30%, level of confidence 95%, and precision 5%) presented by Thrusfield.^16^

**Sample processing and examination. **In each farm and herd, an amount of 25 g fresh fecal samples was collected directly from the rectum of each individual cattle and or calves and fecal smears were prepared. The *Cryptosporidium* oocysts were initially screened using the modified Ziehl-Neelsen staining method.^18^ The parasite species was identified by morphologic and morphometeric criteria which measured at 1000× magnification. Morphologically, *C. parvum* (5.0 × 4.5 µm) oocysts were discriminated from *C. andersoni* oocysts (7.6 × 5.6 µm).^19^

**Molecular procedures. **Since, the modified Ziehl-Neelsen staining is a non-specific staining procedure the PCR procedure was performed to specify genus *Cryptosporidium*. For this purpose, oocysts were purified from fecal specimens of infected cattle using sucrose gradients as described by Arrowood and Sterling ^20^ and subjected to molecular analysis. To rupture *Cryptosporidium* oocysts, 10 times freeze-thaw cycles were performed using liquid nitrogen.^21^ Genomic DNA was extracted by modified phenol-chloroform method using cetyltrimethylammonium bromide (CTAB) at 60 ˚C for 1 hr.^22^

A fragment of 1325 bp of the 18S rRNA gene of *Cryptosporidium* was amplified using two primers (Cryptosense: 5'- TTCTAGAGCTAATACATGCG-3' and Crypto-antisense: 5'- CCCTAATCCTTCGAAACAGGA -3').^23^ PCR reaction was carried out in a 25 µL reaction mixture containing 3 µL of genomic DNA (diluted 1:30), 0.5 µL of *Taq* DNA polymerase (Fermentas, Munich, Germany), 4 µL of 1.25 mM dNTPs (CinnaGen, Tehran, Iran), 1 µL of 50 mM MgCl_2_, 2.5 µL of 10X PCR reaction buffer, 1 µL of each primer (25 µM). The samples were subjected to an initial denaturation step at 94 ˚C for 5 min, followed by 35 cycles of 45 sec at 94 ˚C, 45 sec at 55 ˚C, and 90 sec at 72 ˚C, and a final extension step at 72 ˚C for 7 min. The PCR product was analyzed by electrophoresis on 1.5% (w/v) agarose gel and visualized by staining with 1% ethidium bromide.

**Statistical analysis. **Statistical evaluation was undertaken to compare obtained data with confidence interval of 95% using non-parametric Fisher’s exact test (Version 14.0; SPSS Inc., Chicago, IL, USA). Probability values of *p *< 0.05 were regarded statistically significant.

## Results

The detected *Cryptosporidium* oocysts were nearly spherical in shape and contained four sporozoites. Based on the size, *C. andersoni *oocysts were morphologically distinguishable from* C. parvum* oocysts. The average diameter for *C. parvum* oocysts ranged from 4.5 to 5.0 µm and shape index (SI) as 1.1 ± 0.2, whereas larger *C. andersoni* oocysts ranged from 6.5 to 7.9 µm in size with SI as 1.3 ± 0.4. 

**Fig. 2:**
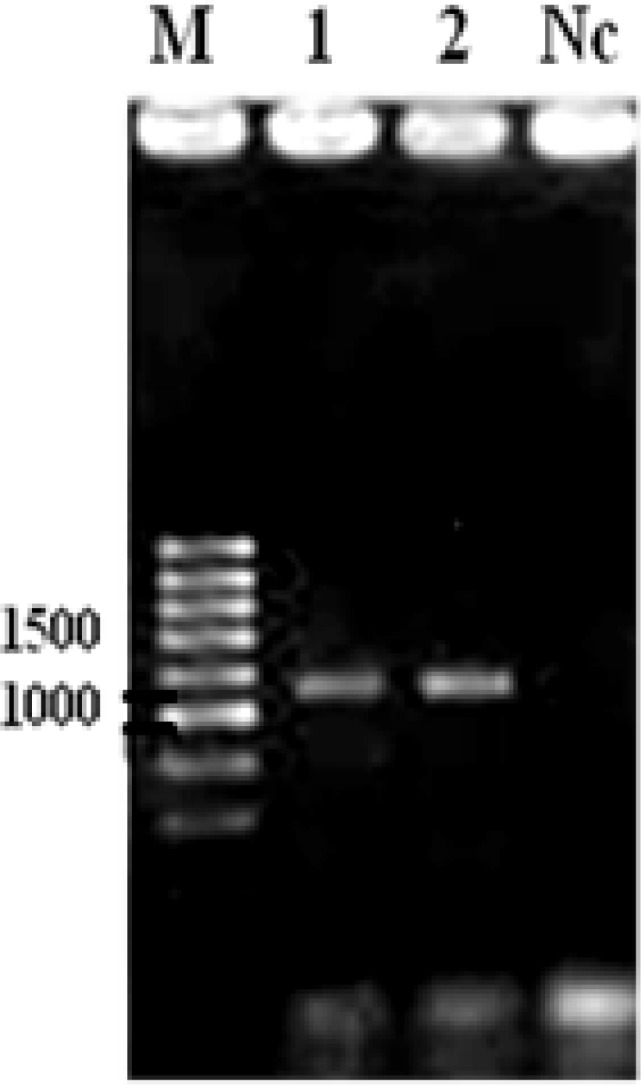
Agarose gel electrophoresis of 18S rRNA PCR products of representative *Cryptosporidium*: Lane 1, positive control; Lane 2, *Cryptosporidium*; Lane Nc, negative control; Lane M, 250 bp DNA size marker

The prevalence of *C. parvum* and *C. andersoni *infection in D or ND cattle and or calves of different age groups have been shown in Table 2. The overall prevalence of *Cryptosporidium *infection was 22.3% (55/246) which confirmed by PCR (Fig. 2). Out of them, 20.3% calves (50/246) and 2.0% cattle (5/246) were infected with *C. parvum* and* C. andersoni,* respectively (Table 2). The prevalence of *Cryptosporidium* infection in calves was significantly higher than that of adult cattle (*p *= 0.025). The prevalence of *Cryptosporidium* infection was also significantly higher in 13.4% (33/246) D than 8.9% (22/246) ND cattle (*p *= 0.025). Among the samples that were positive for *Cryptosporidium* species, 29 out of 246 (11.7%) were from male and 26 out of 246 (10.5%) were from female cattle (*p *= 0.026). The highest prevalence of *C. parvum *infection was found in D calves (13.4%, 33/246), while *C. andersoni* was only detected in ND cattle (8.9%, 22/246) examined in which grazed in plain areas of the region (*p *= 0.0001). *Cryptosporidium parvum* were detected in all examined herds while *C. andersoni* only detected in three herds (18.8%). Mixed infection with both *Cryptosporidium *species was also found in 2.4% (6/246) of infected cattle. There was no significant difference between the prevalence and seasons of investigation (*p *> 0.05).

**Table 1:** Geographical distribution and major risk factors of cattle sampled from Urmia suburban of WAP, Iran

**Study area** **(No. of cattle)**	**Season**	**No.** **of** **herds**	**No. of** **examined** **cattle**	**Fecal consistency**								**Age (year)**				**Sex**		**Geographical** **feature**
				**< 6** *		**> 6 - 3** **		**4 - 6**		**> 6**		**< 6 **	**> 6 - 3 **	**4 - 6 **	**> 6**	**M**	**F**	**Mo Ms P**
				**D**	**ND**	**D**	**ND**	**D**	**ND**	**D **	**ND**							
**Nushan (12)**	**Fall**	1 1 1	4	2	0	0	0	0	1	0	1	2	0	1	1	1	3	+ - -
**Daresanji (8)**			3	1	0	0	1	0	1	0	0	1	1	1	0	2	1	- + -
**Band (16)**			5	1	0	2	0	1	0	0	1	1	2	1	1	2	3	- + -
**Nushan (19)**	**Winter**	1 1 1	8	4	2	1	0	1	0	0	0	6	1	1	0	4	4	+ - -
**Daresanji (21)**			8	4	2	0	1	0	1	0	0	6	1	1	0	3	5	- + -
**Band (23)**			15	7	4	2	1	0	1	0	0	11	3	1	0	11	4	- + -
**Ghasemlu (20)**	**Spring**	1 1 1 1 1 1	10	4	2	0	0	3	0	0	1	6	0	3	1	5	5	+ - -
**Bardasur (29)**			23	11	4	3	0	0	2	0	3	15	3	2	3	14	9	+ - -
**Gharaghaj (33)**			26	16	1	0	0	3	3	0	3	17	0	6	3	12	14	- - +
**Imamkandi (27)**			18	10	0	1	0	1	0	0	6	10	1	1	6	8	10	- - +
**Ziveh (37)**			29	18	2	1	2	0	2	0	4	20	3	2	4	7	22	- - +
**Nazlu (39)**			30	8	0	3	0	6	0	2	11	8	3	6	13	5	25	- - +
**Gojar (25)**	**Summer**	1 1 1 1	17	8	1	0	3	0	0	0	5	9	3	0	5	8	9	- + -
**Zangalan (21)**			16	4	7	0	0	0	0	0	5	11	0	0	5	5	11	+ - -
**Nazlu (18)**			13	3	3	0	2	0	3	0	2	6	2	3	2	6	7	- - +
**Balu (27)**			21	7	9	0	1	0	2	0	2	16	1	2	2	4	17	- - +
**Total**		16	246	108	37	13	11	15	16	2	44	145	24	31	46	97	149	

* **Notes: **month;

** year; D = Diarrheic (soft to diarrheic and or watery feces); ND = Non-diarrheic (normal to semi soft feces); F = Female; M= male; Mo = Mountainous; Ms = Mountain side; P = plain.

**Table 2:** Prevalence (%) of cattle *Cryptosporidium* infection (n = 246) and individual *Cryptosporidium* species from Urmia suburban of WAP, Iran

**Study area** **(No. of cattle)**	**Season**	**Prevalence** ** ( n/N)**	**Fecal consistency**								**Age ** **(year)**				**Sex ** **(%)**		***Cryptosporidium*** ** (%)**	
			**< 6** *		**> 6-3** **		**4-6**		**> 6**		**< 6**	**> 6 - 3**	**4 - 6**	**> 6**	**M**	**F**	***Cp ***	***Ca***
			**D**	**ND**	**D**	**ND**	**D**	**ND**	**D**	**ND**								
**Nushan (12)**	**Fall** ^NSa^	0	0	0	0	0	0	0	0	0	0	0	0	0	0	0	0	0
**Daresanji (8)**		0	0	0	0	0	0	0	0	0	0	0	0	0	0	0	0	0
**Band (16)**		0	0	0	0	0	0	0	0	0	0	0	0	0	0	0	0	0
**Nushan (19)**	**Winter**	1.2	0.8	0	0	0.4	0	0	0	0	0.8	0.4	0	0	1.2	0	1.2	0
**Daresanji (21)**		0	0	0	0	0	0	0	0	0	0	0	0	0	0	0	0	0
**Band (23)**		1.6	0.8	0	0	0	0	0.8	0	0	1.2	0	0.4	0	0.4	1.2	1.6	0
**Ghasemlu (20)**	**Spring**	0	0	0	0	0	0	0	0	0	0	0	0	0	0	0	0	0
**Bardasur (29)**		0	0	0	0	0	0	0	0	0	0	0	0	0	0	0	0	0
**Gharaghaj (33)**		4.0	3.2	0	0	0.8	0	0	0	0	2.4	0.8	0.4	0.4	1.6	2.4	3.2	0.8
**Imamkandi (27)**		0	0	0	0	0	0	0	0	0	0	0	0	0	0	0	0	0
**Ziveh (37)**		3.6	2.4	0	0	1.2	0	0	0	0	2.8	0	0.4	0.4	2.0	1.6	3.2	0.4
**Nazlu (39)**		5.2	2.8	0	0	2.4	0	0	0	0	4.4	0	0	0.8	2.4	2.8	4.4	0.8
**Gojar (25)**	**Summer**	2.0	1.2	0	0	0.8	0	0	0	0	1.6	0	0	0.4	1.2	0.8	2.0	0
**Zangalan (21)**		0	0	0	0	0	0	0	0	0	0	0	0	0	0	0	0	0
**Nazlu (18)**		1.6	0.8	0	0	0.8	0	0	0	0	1.6	0	0	0	1.6	0	1.6	0
**Balu (27)**		2.8	1.2	0	0	1.6	0	0	0	0	2.0	0.8	0	0	1.2	1.6	2.8	0
**Total**		22.3	13.4^Sb^	0	0	8.9	0	0	0	0	17.0^S^^c^	2.4	1.2	2.0	11.7^Sd^	10.5	20.3	2.0

* month;

** year; Ca = *Cryptosporidium andersoni*; Cp =* Cryptosporidium parvum*; n = Number of animals infected with *Cryptosporidium*; N = Total number of examined animals; NS = Non-significant; S = Significant. a (*p *> 0.05); b (*p* = 0.025); c (*p* < 0.0001); d (*p* = 0.026).

## Discussion

Based on morphological characterization described by other researchers,^24^^,^^25^ it was revealed that cattle in the region harbored at least two *Cryptosporidium* species. The small oocysts (mean size: 4.3 ± 0.2 µm) were identified as *C. parvum* (20.3%) and the large ones (average size: 6.8 ± 0.3 µm) were as *C. andersoni* (2.0%). *Cryptosporidium parvum* has been reported to be prevalent in neonates’ worldwide^14^^,^^26^ and was not found in older cattle in any examined herds of the region. Therefore, the higher infection rate of *C. parvum* in current study compared to that of *C. andersoni *appears to reflect the dominance of *C. parvum*. *Cryptosporidium andersoni* has been reported for the first time in Iran by Sohrabi Haghdust.^3^ Thus, this was the first report of *C.* *andersoni* occurrence in cattle of northwestern Iran. In this work, no *C. andersoni* oocysts were detected in cattle < 1 years old, supporting other reports that chronic *C. andersoni* infection usually occurs in adult cattle with no clinical symptoms.^14^^,^^21^^,^^27^ The prevalence of the abomasal species *C.* *andersoni* was reported to be high in adult cattle while it is less pathogenic.^26^^-^^29^

*Cryptosporidium *distribution pattern and prevalence have been reported in many countries throughout the world^25^^,^^30^ and Iran.^31^^,^^32^ The results of the present work revealed that the estimated prevalence was similar to those reported in the previous researches.^14^^,^^33^ Reported *Cryptosporidium *infection prevalence by other researchers varied from 22.0 to 59.0% worldwide^24^ and 3.8 to 42.8% in Iran.^7^^,^^14^^,^^31^^,^^32^ Seasonal changes in prevalence of *Cryptosporidium* infection were observed in spring and summer in the region. However, there was no significant association. In India, the highest prevalence of *Cryptosporidium* infection in cattle was reported in rainy season followed by summer and winter (*p *< 0.01).^34^ These trends may reflect direct zoonotic contact and indirect effects of rainfall, farming events such as calving, and environmental pollution with farm waste.^2^

In the present study, the age of examined cattle had significant effect on the prevalence. In addition, the prevalence of *C. parvum* was decreased with increasing age. The age related distribution of *C. parvum* infection in this age group was similar to that previously reported in cattle of other parts of Iran.^14^^,^^32^ *Cryptosporidium parvum* has been also reported to primarily infect D young calves and shed the specie.^26^^,^^30^ It seems that ND older cattle with low prevalence and without clinical symptoms of cryptosporidiosis may serve as carriers for young calves with an immature immune system in the region. *Cryptosporidium *infection in this study was considered to be a probable cause of diarrhea in neonates as significant association was found in previous studies.^30^^,^^35^^,^^36^ Fotouhi Ardekani *et al.* found significant difference between D (31.8%) and ND (17.4%) conditions.^14^ Also, Brook *et al.* noted that age was correlated with consistency of the feces ^30^ so that in younger animals, feces are tending to be looser due to the liquid nature of the milk diet. Sex of examined cattle in the present study had also significant association with the prevalence. This finding was in concordance with previous research by Radfar *et al.*^32^

The findings described in this investigation suggested that *C. parvum *is the most common species in cattle and farms should be also considered as a potential source of surface water contamination. Thus, further investigations may reveal more information about economic effects of this parasite and public health concern in the region. Furthermore the source of infection should be investigated and control measures should be established in the future. 
